# Identification of novel methylation markers in HPV-associated oropharyngeal cancer: genome-wide discovery, tissue verification and validation testing in ctDNA

**DOI:** 10.1038/s41388-020-1327-z

**Published:** 2020-05-15

**Authors:** Kiyoshi Misawa, Atsushi Imai, Hirotaka Matsui, Akinori Kanai, Yuki Misawa, Daiki Mochizuki, Masato Mima, Satoshi Yamada, Tomoya Kurokawa, Takuya Nakagawa, Hiroyuki Mineta

**Affiliations:** 1 Department of Otolaryngology/Head and Neck Surgery, Hamamatsu University School of Medicine, Shizuoka, Japan; 2 Department of Molecular Laboratory Medicine, Graduate School of Medical Sciences, Kumamoto University, Kumamoto, Japan; 3 Research Institute for Radiation Biology and Medicine, Hiroshima University, Hiroshima, Japan; 4 Department of Otorhinolaryngology/Head and Neck Surgery, Graduate School of Medicine, Chiba University, Chiba, Japan

**Keywords:** Head and neck cancer, DNA methylation, DNA methylation

## Abstract

Human papilloma virus (HPV)-associated oropharyngeal cancer (OPC) is an independent tumour type with regard to cellular, biological, and clinical features. The use of non-invasive biomarkers such as circulating tumour DNA (ctDNA) may be relevant in early diagnosis and eventually improve the outcomes of patients with head and neck squamous cell carcinoma (HNSCC). Genome-wide discovery using RNA sequencing and reduced representation bisulfite sequencing yielded 21 candidates for methylation-targeted genes. A verification study (252 HNSCC patients) using quantitative methylation-specific PCR (Q-MSP) identified 10 genes (*ATP2A1*, *CALML5*, *DNAJC5G*, *GNMT*, *GPT*, *LY6D*, *LYNX1*, *MAL*, *MGC16275*, and *MRGPRF*) that showed a significant increase recurrence in methylation groups with OPC. Further study on ctDNA using Q-MSP in HPV-associated OPC showed that three genes (*CALML5*, *DNAJC5G*, and *LY6D*) had a high predictive ability as emerging biomarkers for a validation set, each capable of discriminating between the plasma of the patients from healthy individuals. Among the 42 ctDNA samples, methylated *CALML5*, *DNAJC5G*, and *LY6D* were observed in 31 (73.8%), 19 (45.2%), and 19 (45.2%) samples, respectively. Among pre-treatment ctDNA samples, methylated *CALML5*, *DNAJC5G*, and *LY6D* were observed in 8/8 (100%), 7/8 (87.5%), and 7/8 (87.5%) samples, respectively. Methylated *CALML5*, *DNAJC5G*, and *LY6D* were found in 2/8 (25.0%), 0/8 (0%), and 1/8 (12.5%) of the final samples in the series, respectively. Here, we present the relationship between the methylation status of three specific genes and cancer recurrence for risk classification of HPV-associated OPC cases. In conclusion, ctDNA analysis has the potential to aid in determining patient prognosis and real-time surveillance for disease recurrences and serves as an alternative method of screening for HPV-associated OPC.

## Introduction

Head and neck squamous cell carcinoma (HNSCC) prognoses remain poor due to high recurrence rates [1]. The prognosis of HNSCC is largely based on pathological staging systems. Current post-treatment surveillance of HNSCC patients is monitored via clinical evaluation combined with flexible endoscopy and conventional imaging [2]. Genomic sequencing has delivered vast amounts of information regarding different cancer subtypes and is providing new therapeutic targets [3]. The hope is that effective and precise theragnostic-based options will provide medical benefit to HNSCC patients.

The incidence of human papilloma virus (HPV)-associated oropharyngeal cancer (OPC) has increased in recent decades and is now the most prevalent HPV-related cancer in developed countries [4]. An estimated 70–80% of OPCs are now attributed to HPV in the United States, western Europe and Japan [5]. Compared with smoking-related or alcohol-related OPC, HPV positivity is associated with a better prognosis [6]. Despite the prognostic value added by HPV status, subsets of patients continue to demonstrate outcomes discordant with their disease stage. Several studies have demonstrated that risk stratification of patients with HPV-associated OPC is caused by tobacco smoking history: low risk (HPV-associated, 10 pack years) and intermediate risk (HPV-associated, >10 pack years) [7–9]. Previous studies have suggested that the combination of HPV DNA and p16 testing was a significantly better prognostic marker than either HPV DNA or p16 alone [10].

Recently, the potential usefulness of circulating tumour DNA (ctDNA) as a biomarker was shown in several applications in cancer management. A liquid biopsy utilising tumour DNA found in blood could address many limitations of tissue biopsies, while also opening the innovative paradigms in cancer care. Analyses using DNA methylation-based strategies for detection of ctDNA have suggested that such approaches may provide new advance for early cancer diagnosis [11]. As ctDNA is composed largely of short fragments, short amplicons are required for maximum sensitivity of PCR reactions, particularly if mutations are being detected [12]. Recent studies highlight this potential for ctDNA methylation, demonstrating that even with cancer-specific epigenetic changes, sufficient cell-type specificity remains to be determined to permit identification of cell-of-origin [13].

To our knowledge, our study is the first to suggest that Calmodulin-like 5 (*CALML5*), DnaJ heat shock protein family member C5 gamma (*DNAJC5G*), and Lymphocyte antigen 6 complex locus D (*LY6D*) methylation is associated with worse disease-free survival (DFS) and may be a critical event in HPV-associated OPC. To improve the survival rate for head and neck cancer, real-time monitoring of patients following surgery as a measure of therapeutic efficacy can be carried out. Here, we report the results of a combined discovery, verification, and validation study aimed at identifying novel blood-based DNA methylation markers, and document their ability to efficiently improve the diagnosis and prognosis of HPV-associated OPC.

## Results

### Experimental setup of the study and genome-wide epigenetic profiling using RNA sequencing (RNA-seq) and reduced representation bisulfite sequencing (RRBS)

**Fig. 1:**
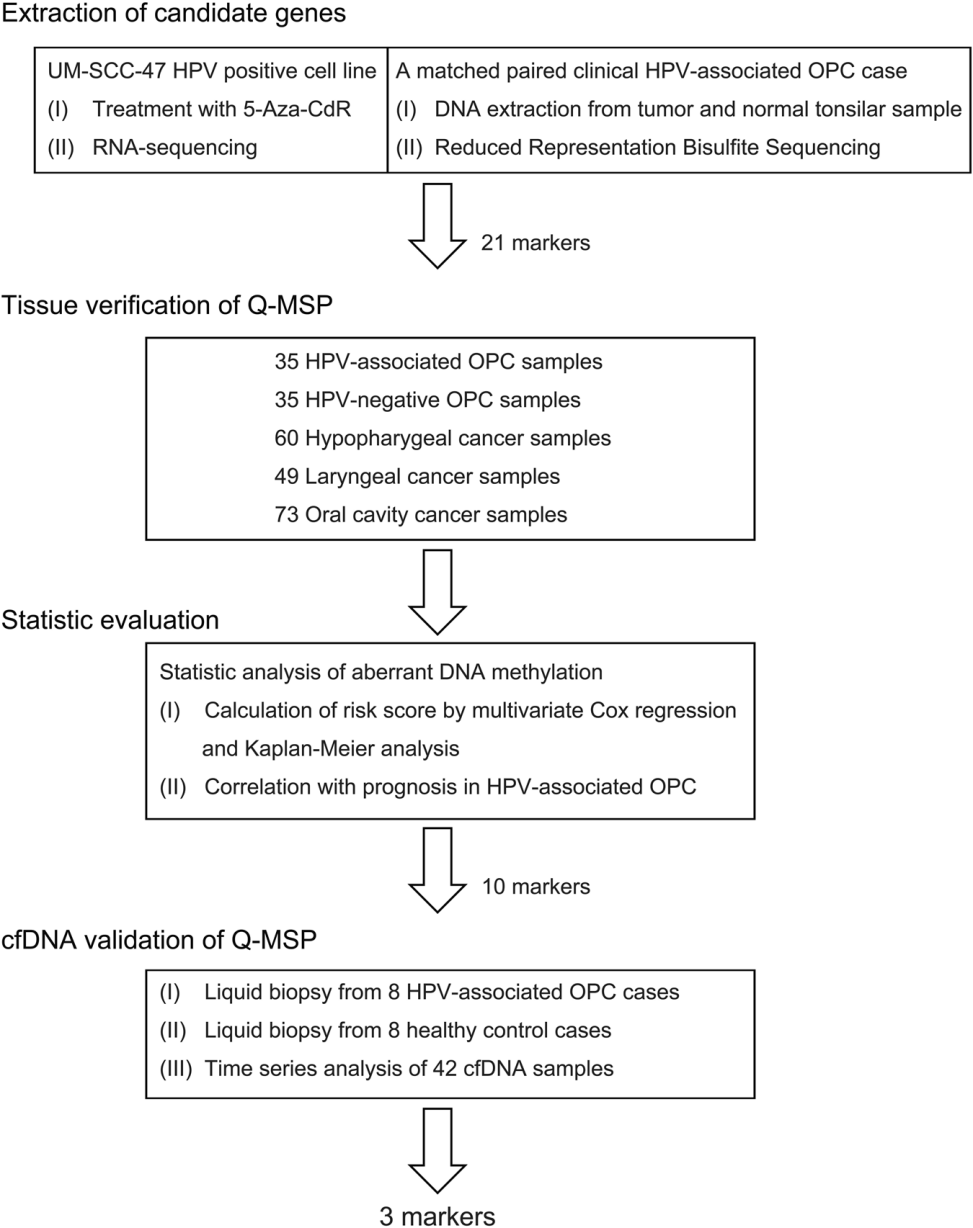
Flowchart of the marker selection procedures. Design of the study; discovery of epigenetic markers of HPV-associated cell lines and HPV-associated OPC patients.

An outline of the study is given in Fig. 1. A total of 1,968 markers were upregulated at least two-fold after 5-aza-2′-deoxycytidine (5-Aza-CdR) treatment compared to untreated controls identified using RNA-seq. In the RRBS data, we selected 284 hypermethylated promoter regions with more than a 20-fold difference between the tumour and normal tonsillar samples. By combining the RNA-seq data with the RRBS data, we identified 21 possibly methylated markers for HPV-associated OPC. The findings from the discovery profiling, *AGAP2*, *ALPL*, *ANGPTL2*, *ATP2A1, CALML5, DNAJC5G, FDFT1, GNMT, GPT, HOXB3, KLK11, LMF1, LY6D, LYNX1, MAL, MGC16275, MRGPRF, NKPD1, SH2D3C, TNNI2*, and *ZNF876P*, were further analysed using quantitative methylation-specific PCR (Q-MSP) in an independent large series of HNSCC specimens.

### Verification analysis of methylation status in HNSCC tissue samples

**Fig. 2:**
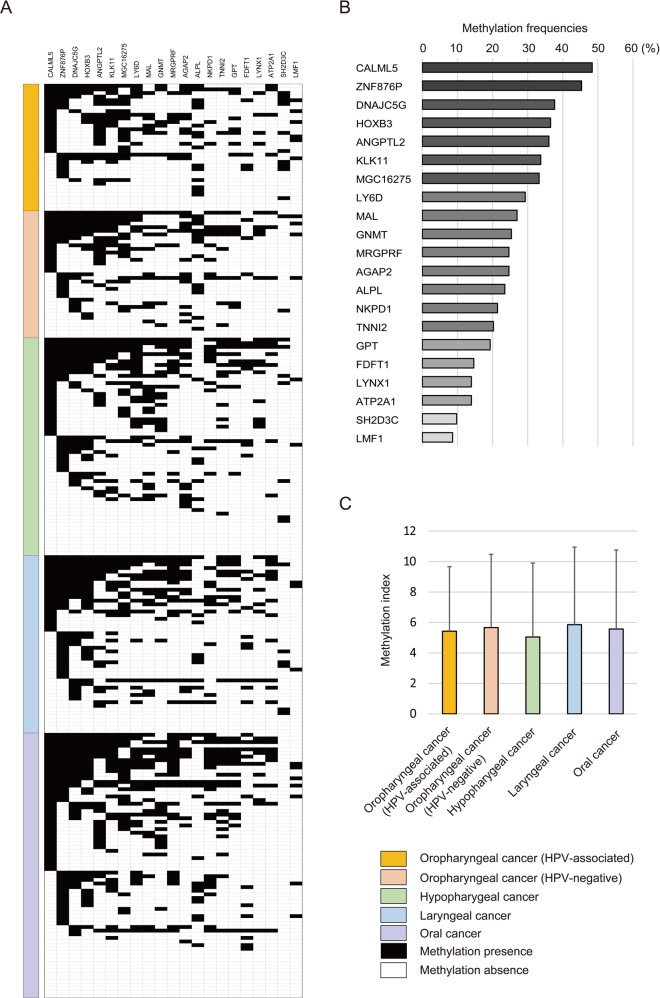
Site-specific methylation frequencies for 21 epigenetic marker genes. **a** Comparison of methylation states of the promoters of the 21 epigenetic marker genes in patients with (HPV-associated and HPV-negative) oropharyngeal, hypopharyngeal, laryngeal, or oral cancer. **b** Bar graph showing the methylation frequencies of the 21 genes. **c** The mean methylation index values for various groups were compared using Student’s *t* tests.

A Q-MSP analysis of the methylation status of 21 epigenetic marker genes was performed using 252 primary HNSCC samples, including 35 HPV-associated OPC, 35 HPV-negative OPC, 60 hypopharyngeal cancers, 49 laryngeal cancers, and 73 oral cavity cancer samples (Fig. 2a). The methylation frequencies for these genes are summarised in Table 1 and Fig. 2b. Site-specific methylation frequencies across the 21 genes for the oropharynx, hypopharynx, larynx, and oral cavity are shown in Fig. 2c. There were no significant associations between the methylation index and the primary tumour site.

**Table 1:** Correlation between primary tumor sites and methylation status.

Genes	Methylation status	Oropharyngeal cancer		Hypopharygeal cancer (*N* = 60)	Laryngeal cancer (*N* = 49)	Oral cavity cancer (*N* = 73)	*P* values*			
		HPV-associated (*N* = 35)	HPV-negative (*N* = 35)				HPV+ vs HPV-	HPV+ vs Hy	HPV+ vs La	HPV+ vs Or
AGAP2	Methylated	6	4	14	15	23				
	Unmethylated	29	31	46	34	50	0.734	0.604	0.204	0.163
ALPL	Methylated	15	13	7	10	14				
	Unmethylated	20	22	53	39	59	0.636	<0.001*	0.031*	0.011*
ANGPTL2	Methylated	18	15	18	16	24				
	Unmethylated	17	20	42	33	49	0.485	0.049*	0.114	0.091
ATP2A1	Methylated	4	5	10	6	10				
	Unmethylated	31	30	50	43	63	1	0.562	1	1
CALML5	Methylated	19	17	27	21	38				
	Unmethylated	16	18	33	28	35	0.811	0.403	0.376	0.840
DNAJC5G	Methylated	8	15	21	23	28				
	Unmethylated	27	20	39	26	45	0.083	0.253	0.038*	1
FDFT1	Methylated	4	3	8	9	13				
	Unmethylated	31	32	52	40	60	0.714	1	0.543	0.573
GNMT	Methylated	6	7	17	13	21				
	Unmethylated	29	28	43	36	52	1	0.320	0.428	0.239
GPT	Methylated	7	7	10	10	15				
	Unmethylated	28	28	50	39	58	1	1	1	1
HOXB3	Methylated	9	14	20	23	26				
	Unmethylated	26	21	40	26	47	0.308	0.494	0.068	0.381
KLK11	Methylated	14	13	15	18	25				
	Unmethylated	21	22	45	31	48	0.811	1	1	1
LMF1	Methylated	6	4	2	3	7				
	Unmethylated	29	31	58	46	66	0.734	0.048*	0.154	1
LY6D	Methylated	8	9	22	13	22				
	Unmethylated	27	26	38	36	51	0.789	0.178	0.800	0.496
LYNX1	Methylated	4	3	7	12	9				
	Unmethylated	31	32	53	37	64	0.714	1	0.165	1
MAL	Methylated	7	9	20	13	19				
	Unmethylated	28	26	40	36	54	0.585	0.238	0.606	0.632
MGC16275	Methylated	16	14	16	14	24				
	Unmethylated	19	21	44	35	49	0.116	0.620	0.448	0.182
MRGPRF	Methylated	10	12	15	14	11				
	Unmethylated	25	23	45	35	62	0.618	1	1	1
NKPD1	Methylated	3	8	11	10	22				
	Unmethylated	32	27	49	39	51	0.116	0.241	0.221	0.014*
SH2D3C	Methylated	10	2	7	6	0				
	Unmethylated	25	33	53	43	73	0.013*	0.052	0.090	<0.001*
TNNI2	Methylated	2	8	13	9	19				
	Unmethylated	33	27	47	40	54	0.048*	0.045*	0.111	0.017*
ZNF876P	Methylated	14	16	22	28	34				
	Unmethylated	21	19	38	21	39	0.639	1	0.129	0.542

Chi square test used to calculate *p* value.

*HPV+ vs HPV-* HPV-associated oropharyngeal cancer vs HPV-negative oropharyngeal cancer, *Hy* Hypopharyngeal cancer, *La* Laryngeal cancer, *Or* Oral cavity cancer.

*p < 0.05 considered statistically significant, the same as below.

### Association between methylation index and clinicopathological characteristics

**Fig. 3:**
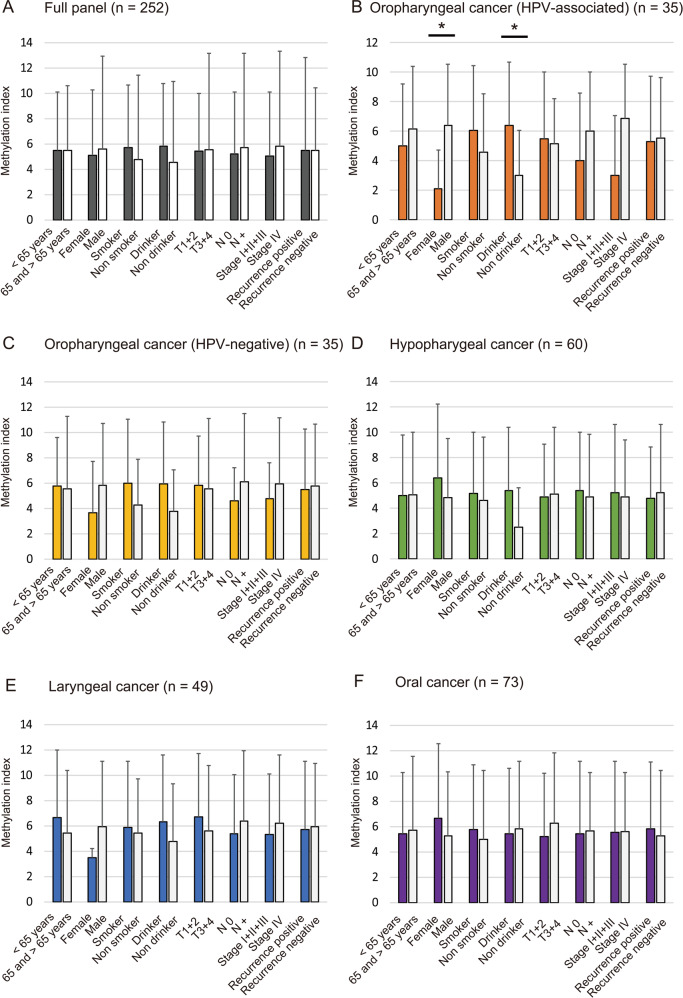
Association between methylation index and selected clinical parameters. Mean methylation index for the various groups compared using Student’s *t* test. Associations between methylation index and selected epidemiologic and clinical characteristics: **a** Among the 252 cases; no differences were noted with regard to any of the clinical characteristics; **b** HPV-associated OPC; statistically significant relationships were found between the methylation index and gender or alcohol consumption; **c** HPV-negative oropharyngeal cancer; **d** hypopharyngeal cancer; **e** laryngeal cancer; **f** oral cancer. Means and standard deviations are indicated, and statistical comparisons between groups are depicted. *P < 0.05 was considered to represent a statistically significant difference.

For the full panel of HPV-negative oropharyngeal, hypopharyngeal, laryngeal, and oral cancer patients, there were no significant associations with any clinicopathological characteristics (Fig. 3a–f). Among the HPV-associated OPC, the methylation index was significantly lower in females than in male patients, as well as in non-drinkers relative to drinkers (Fig. 3b).

### Stratification analysis

**Fig. 4:**
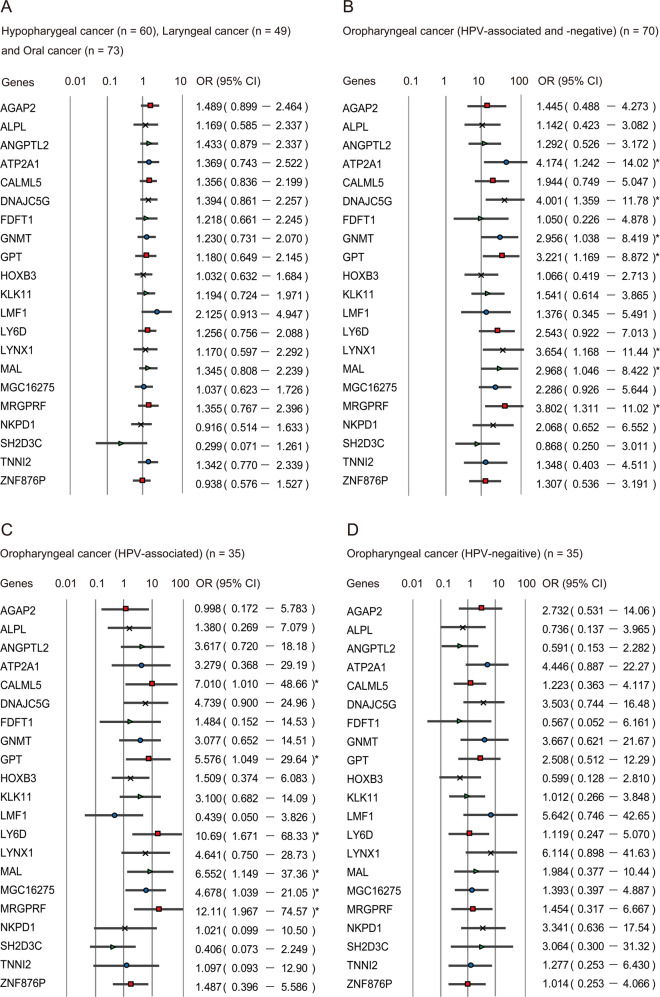
Risk of recurrence based on methylation states of 21 genes in tumours with different origins. Odds ratios for recurrence were determined using a Cox proportional hazards model adjusted for age (≥65 vs. <65 years), gender, smoking status, alcohol intake, and tumour stage (I–II vs. III–IV). **a** Hypopharygeal, laryngeal and oral cancer (*n* = 182), **b** HPV-associated OPC and HPV-negative OPC (*n* = 70), **c** HPV-associated OPC (*n* = 35), **d** HPV-negative OPC (*n* = 35). OR: odds ratio. CI confidence interval. *P < 0.05.

The relation between the methylation status and risk of recurrence was analysed through multivariate analysis using a Cox proportional hazards regression model adjusted for age, gender, smoking status, alcohol consumption, and clinical tumour stage. The methylation of the 21 marker genes was not associated with recurrence in patients with hypopharyngeal, laryngeal, or oral cancers (Fig. 4a). In patients with oropharyngeal cancers (HPV-associated and HPV-negative), we found that hypermethylation of *ATP2A1*, *DNAJC5G*, *GNMT*, *GPT*, *LYNX1*, *MAL*, and *MRGPRF* were associated with significantly reduced DFS, with hazard ratios of 4.17 (95% confidence interval (CI), 1.24–14.02), 4.00 (95% CI: 1.36–11.78), 2.96 (95% CI: 1.04–8.42), 3.22 (95% CI: 1.17–8.87), 3.65 (95% CI: 1.17–11.44), 2.97 (95% CI: 1.05–8.42), and 3.80 (95% CI: 1.31–11.02), respectively (Fig. 4b). In patients with HPV-associated OPC, hypermethylation of the *CALML5*, *GPT*, *LY6D*, *MAL*, *MGC16275*, and *MRGPRF* genes was associated with a significantly reduced survival, with hazard ratios of 7.01 (95% CI: 1.01–48.66), 5.58 (95% CI: 1.05–29.64), 10.69 (95% CI: 1.67–68.33), 6.55 (95% CI: 1.15–37.36), 4.68 (95% CI: 1.04–21.05), and 12.1 (95% CI: 1.97–74.57), respectively (Fig. 4c). In patients who had HPV-negative OPC, the methylation of the 21 marker genes was associated with an elevation in the odds of recurrence that was not significant (Fig. 4d).

### Kaplan–Meier estimates in HPV positive oropharyngeal cancer patients

**Fig. 5:**
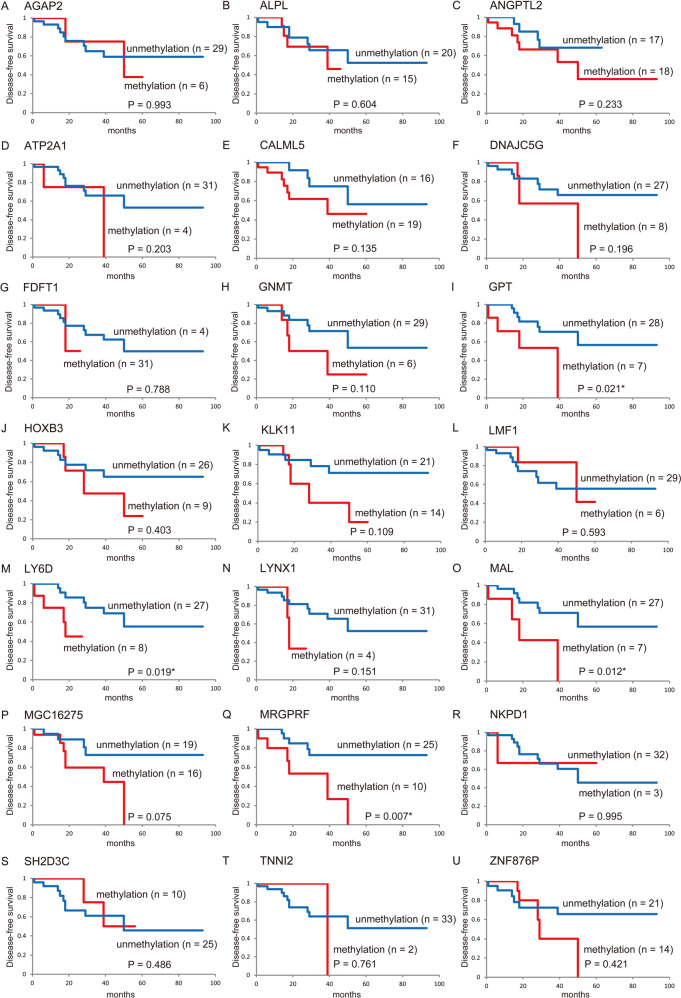
Kaplan–Meier survival curves for the 35 HPV-associated oropharyngeal cancer patients according to the methylation status of the 21 target genes. DFS for (**a**) *AGAP2*, (**b**) *ALPL*, (**c**) *ANGPTL2*, (**d**) *ATP2A1*, (**e**) *CALML5*, (**f**) *DNAJC5G*, (**g**) *FDFT1*, (**h**) *GNMT*, (**i**) *GPT*, (**j**) *HOXB3*, (**k**) *KLK11*, (**l**) *LMF1*, (**m**) *LY6D*, (**n**) *LYNX1*, (**o**) *MAL*, (**p**) *MGC16275*, (**q**) *MRGPRF*, (**r**) *NKPD1*, (**s**) *SH2D3C*, (**t**) *TNNI2*, and (**u**) *ZNF876P* in the case of methylated (red lines) and unmethylated (blue lines) genes. A probability of < 0.05 (*P < 0.05) was considered statistically significant.

Based on the HPV-associated OPC patients, Kaplan–Meier survival curves for each of the 21 genes are shown in Fig. 5. The DFS time did not significantly differ between patients with methylated genes and those with unmethylated genes, with four notable exceptions: DFS was significantly shorter when *GPT*, *LY6D*, *MAL*, and *MRGPRF* were methylated (*P* = 0.021, *P* = 0.019, *P* = 0.012, and *P* = 0.007, respectively; Fig. 5).

### Validation analysis of methylation status in clinical primary samples, paired ctDNA samples and healthy ctDNA samples

**Fig. 6:**
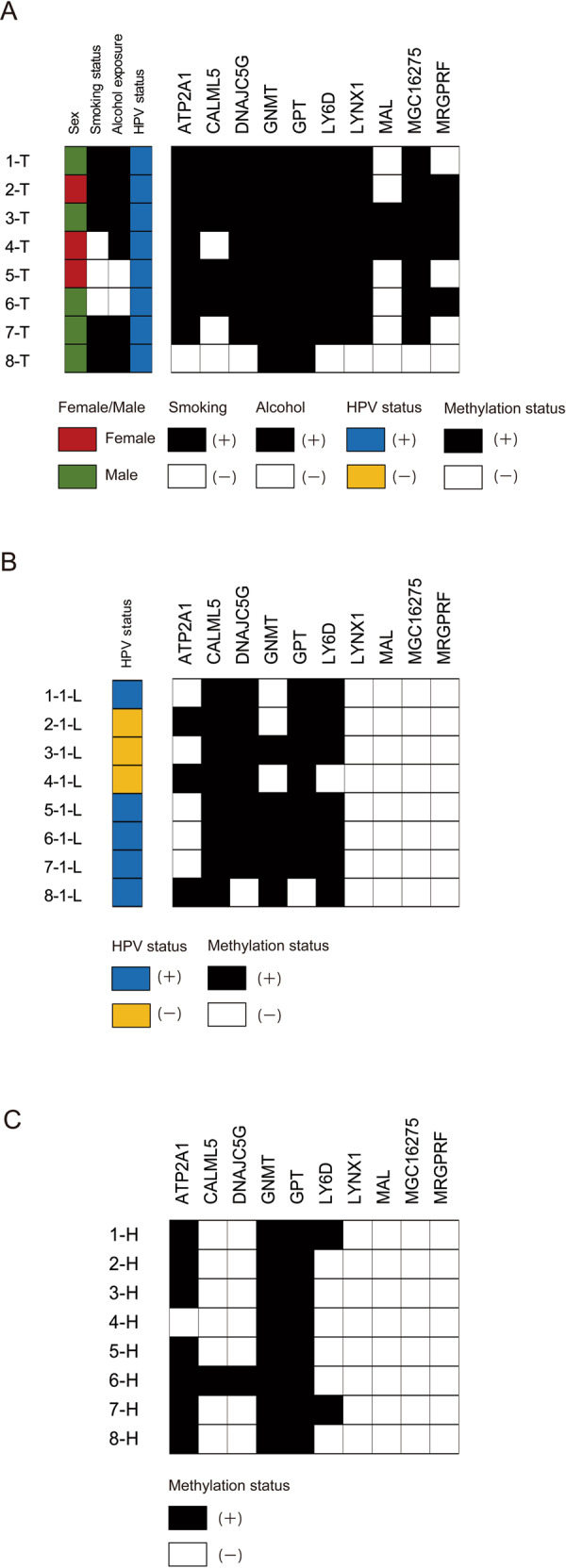
Validation analysis of methylation status in clinical primary samples, paired ctDNA samples, and healthy ctDNA samples. Comparison of the methylation states of 10 markers in (**a**) 8 primary samples, (**b**) ctDNA isolated from matched plasma samples from the same patients as above and (**c**) 8 healthy individuals.

A prognostic risk category based on the methylation status of *ATP2A1*, *CALML5*, *DNAJC5G*, *GNMT*, *GPT*, *LY6D*, *LYNX1*, *MAL*, *MGC16275*, and *MRGPRF* refined the risk stratification for outcomes as an independent prognostic factor for HPV-associated OPC. We found a concordance between the primary samples and matched ctDNA in HPV-associated OPC for *ATP2A1*, *CALML5*, *DNAJC5G*, *GNMT*, *GPT*, and *LY6D* promoter methylation but not for *LYNX1*, *MAL*, *MGC16275*, and *MRGPRF* (Fig. 6a, b). We further compared the methylation of these 10 markers in eight healthy individuals. The methylation status of *ATP2A1*, *GNMT*, and *GPT* was 7/8 (87.5%), 8/8 (100%) and 8/8 (100%), respectively (Fig. 6c). Through validation testing, we selected three genes (*CALML5*, *DNAJC5G*, and *LY6D*), which could distinguish HPV-associated OPC patients from a healthy control population.

### Serial ctDNA methylation analysis

**Fig. 7:**
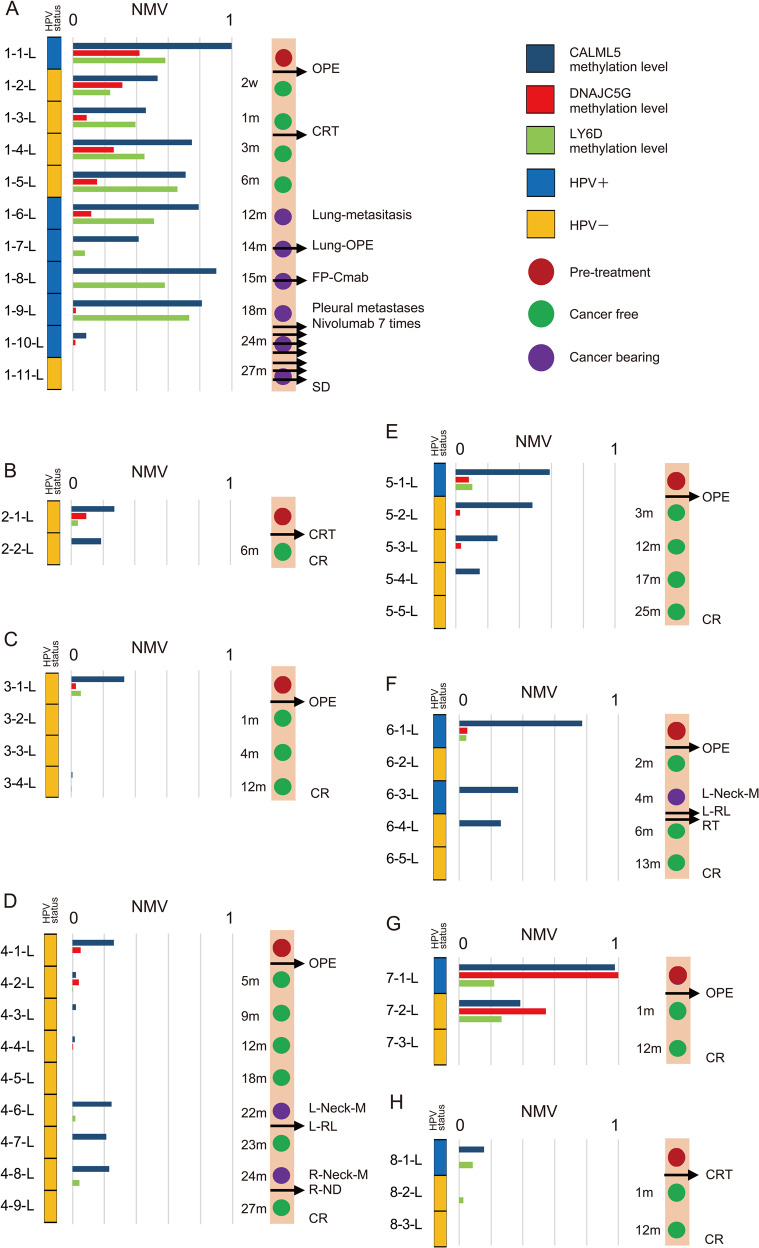
Methylation status for each patient in serial ctDNA samples of HPV-associated OPC patients receiving treatment at different time points during follow-up. **a** Patient 1, initial treatment was a right oropharyngectomy and bilateral ND. Concomitant CRT was administered postoperatively. During follow-up, the patient was also found to have fluorodeoxyglucose-avid lung nodules as well, one of which was subsequently extracted, confirming metastatic HPV16 positive SCC. After one dose of 5-FU+ CDDP+ cetuximab and seven doses of nivolumab, the clinical response was a stable disease (SD) during chemotherapy. After initial treatment, three ctDNA methylation markers were non-decreasing. However, three ctDNA methylation levels were showing a significant decrease due to the response to the chemotherapy. **b** Patient 2 experienced a reduction in ctDNA methylation levels after concurrent CRT. **c** Patient 3 represented a drop in ctDNA methylation levels after oropharyngectomy and bilateral ND. **d** Patient 4, *CALML5* and *DANJC5G* methylation levels gradually decreased after oropharyngectomy and a left modified ND. In this case, the elevation of *CALML5* and *LY6D* methylation levels correlated with regional lymph node metastasis. **e** Patient 5, ctDNA methylation levels gradually decreased after oropharyngectomy and a left modified ND. **f** Patient 6 experienced a reduction in ctDNA methylation levels after oropharyngectomy and a left ND. In this case, *CALML5* methylation level enhances metastasis of regional lymph node. **g** Patient 7 was treated by an excision of the tonsillar tumour with bilateral modified ND. After 12 months, the three ctDNA methylation markers were undetectednot detected. **h** Patient 8, received concurrent CRT and, experienced a reduction in ctDNA methylation levels.

The interrelationship between clinical evaluations and changes in serial ctDNA methylation levels of *CALML5*, *DNAJC5G*, and *LY6D* in samples from Patients 1–8 appears in Fig. 7a–h. Sixty-three percent of cases (5/8) had detectable ctHPVDNA in their pre-treatment liquid biopsy samples. Three cases had been diagnosed with disease recurrence: one lung metastasis case and two delayed neck lymph node metastases cases. *CALML5* methylation was observed in 31/42 (73.8%) of all samples, in 8/8 (100%) pre-treatment samples, in 8/9 (88.9%) cancer-bearing samples, and in 2/8 (25.0%) of the final samples in the series. *DNAJC5G* was methylated in the ctDNA samples of 19/42 (45.2%), 7/8 (87.5%), 2/9 (22.2%), and 0/8 (0%) for all samples, pre-treatment samples, cancer-bearing samples, and the final samples in the series, respectively. The *LY6D* was methylated in 21/42 (50.0%), 7/8 (87.5%), 6/9 (66.6%), and 1/8 (12.5%) of all samples, pre-treatment samples, cancer-bearing samples, and the final samples in the series, respectively. The changes in ctDNA methylation are, thus, associated with the clinical condition.

## Discussion

The biomarkers we describe have the potential to affect one of the most important unmet clinical needs in HPV-associated OPC: a risk classification system that is more effective than patient history and physical examination [14]. The advantages of our study are the evaluation of a large, independent series of different patient-derived sample types, and the use of a discovery–verification–validation approach, which allowed us to evaluate the succession of methylation events and the selection of methylation targets which are prognostically relevant and associated with HPV-associated carcinogenesis.

Plasma circulating tumour HPV DNA (ctHPVDNA) is a promising biomarker for monitoring treatment responses in patients with HPV-associated OPC [15]. According to the results, the patients with ctHPVDNA OPC possessed significantly higher HPV DNA viral loads in their tumour tissues than the ctHPVDNA-negative patients [16]. Longitudinal monitoring of ctHPVDNA during post-treatment surveillance could accurately detect clinical disease recurrence [17]. The detection of small tumours indicates that ctHPVDNA could be readily used in early detection screening [18].

Tumour DNA, as determined by the presence of somatic mutations (TP53: 72%, p16 (*CDKN2A*): 22%, p110a (*PIK3CA*): 21%, and Notch Receptor 1 (*NOTCH1*): 19%) is highly specific for HNSCC in The Cancer Genome Atlas (TCGA) data [19]. The use of mutations commonly found in cancers is clinically attractive, and quantification of *TP53* mutations in ctDNA from HNSCC patients using digital PCR is technically feasible [20]. *PIK3CA* mutation was detected in the plasma samples of 31% HNSCC patients [21]. The detection of early diagnosis markers becomes slightly more challenging when the number of alterations need to be detected across large multi-exon genes, such as *TP53* or *NOTCH1* [22].

HNSCC is characterised by some early epigenetic alterations, several of these being biomarkers found in circulation [23]. DNA methylation of certain genes in other cancers sites can be found among the ctDNA and has been studied by many groups, but it should be noted that substantial numbers of healthy controls also have methylation of ctDNA for these genes [24, 25]. At present, there are only a few studies which investigated a relation between ctDNA methylation events and prognosis in HNSCC [26, 27]. Quantitative DNA methylation levels for septin 9 (*SEPT9*) and short-stature homeobox 2 (*SHOX2*) in the ctDNA proved to be powerful prognostic and molecular staging biomarkers for identifying patients at a higher risk of tumour recurrence [26]. Hypermethylation of endothelin receptor type B (*EDNRB*) was identified in the serum of 10% of patients with HNSCC with a high degree of specificity [27].

By unbiased comprehensive stepwise verification and validation studies, we identified three new promising methylation markers (*CALML5*, *DNAJC5G* and *LY6D*) associated with HPV-associated OPC. To the best of our knowledge, methylation of *CALML5* and *DNAJC5G* have not been described before in HNSCC, whereas *LY6D* was identified previously [28].

*CALML5*, a skin-specific calcium binding protein, is closely associated with keratinocyte differentiation [29]. CALML5 protein is a regulator of terminal differentiation of epidermal cells, and high-density CALML5 conditions promote YAP1 translocation to the cytoplasm [30]. *CALML5* ubiquitination in the nucleus is involved in the carcinogenesis of breast cancer in young women [31].

The human genome encodes more than 40 identified DNAJ protein family members, which can be classified into three groups (A, B, and C) according to their domain structures. Cysteine string protein (CSP), a member of the DnaJ/heat shock protein 40 (Hsp40) family, is comprised of three CSP peptides (α, β and γ) that are encoded by the DNAJC5A, B and G genes. Distinct HSP40 family members regulate the replication and pathogenesis of various viruses [32]. DNAJC5G significantly inhibits the replication efficiencies of adenovirus, vaccinia virus, and HIV-1 [33]. Another member of the HSP40 proteins, DNAJA3, was initially characterised through its interaction with the HPV16 E7 oncoprotein [34].

LY6D is a member of the LY6 family, and it is a membrane-bound protein with a glycosylphosphatidylinositol anchor [35]. LY6D plays an important role in the adhesion of head and neck cancer cells to endothelial cells, and is detected of micrometastases in lymph nodes of patients with HNSCC [28]. LY6D may serve as a prognostic maker for advanced prostate cancer and oestrogen receptor-positive breast carcinomas [36, 37]. Recently, aberrant DNA hypermethylation in the *LY6D* gene promoter region has been reported in several tumours such as a gefitinib-resistant lung cancer cell line and helicobacter-associated gastric cancer in mice [38, 39].

Liquid biopsy needs further methodological evaluation as well as cost-based assessment [40]. A smooth translation into clinical practice requires a systematic assessment of the health economic benefits [41]. Patients who have undetectable ctHPVDNA during clinical follow-up are unlikely to have recurrent disease and may be spared routine radiographic and in-office pharyngoscopic surveillance [17]. Furthermore, ctHPVDNA surveillance may be more cost effective than current surveillance approaches (e.g., PET-CT) [15].

Currently, the lack of an effective OPC screening program is because there are no identified OPC precursor lesions. The HPV-associated carcinogenesis initially arises in tonsillar crypts, which may be the most likely reason why the HPV prevalence in tonsillar cells is higher [4]. In the future, we think that highly sensitive and tissue-specific ctDNA biomarkers of HPV-associated OPC will be discovered, allowing early detection. This study, involving human specimens and high-throughput profiling platforms, may be susceptible to measurement bias from various sources; accordingly, the use of methylation markers in clinical practice requires further testing in prospective studies with larger HNSCC cohorts. Eventually, these efforts will lead to the identification of new oncological biomarkers for early detection and outcome prediction, which is a prerequisite for realising the advantages of precision medicine. Our study demonstrated the utility of using parallel serial assessment of ctDNA methylation in the treatment evaluation of HPV-associated OPC.

## Materials and methods

### Cell lines and treatments

The UM-SCC-47 cell line (HPV16 positive), derived from a primary tumour of the lateral tongue of a male patient, was obtained from the University of Michigan (Ann Arbor, MI, USA). The cells were cultured in Dulbecco’s modified Eagle’s medium (FUJIFILM Wako Pure Chemical Corporation, Osaka, Japan) supplemented with 10% foetal bovine serum (Gibco, Thermo Fisher Scientific Inc., Waltham, MA) and 1% penicillin/streptomycin (Wako) in a humidified atmosphere containing 5% CO_2_ at 37 °C. Twelve hours after plating, cultures were incubated either for 48 h with 5-Aza-CdR (5 μM; Sigma, St. Louis, MO). The medium was then removed, and cultures were maintained in standard Dulbecco’s modified Eagle’s medium, which was replaced every other day.

### Clinical tumour samples

Surgical HNSCC tumour and matched adjacent non-tumour tissues were obtained from 252 patients who underwent surgical resection at the Department of Otolaryngology/Head and Neck Surgery, Hamamatsu University School of Medicine (Hamamatsu, Shizuoka, Japan). Written informed consent was obtained from individual patients before surgery and the experimental protocol was approved by the Hamamatsu University School of Medicine (date of board approval: ethics code: 25–149 and 17–041). The ratio of males to females was 217:35. The mean age was 65.0 years (range, 32–92 years). Primary tumours were composed of 35 HPV-associated OPC, 35 HPV-negative OPC, 60 hypopharyngeal carcinomas, 49 laryngeal carcinomas, and 73 oral cavity carcinomas (Supplementary Table S1).

### Liquid biopsy samples

ctDNA was isolated from 4.0 mL of blood plasma by affinity-based binding to magnetic beads according to the manufacturer’s instructions (QIAamp MinElute ccfDNA Kit, QIAGEN). Peripheral blood (10 ml each) was collected in cell-stabilising tubes (Cell-Free DNA Collection Tube, Roche, CA, USA). ctDNA was extracted on the day of surgery or outpatient visits. Eight patients with HPV-associated OPC and eight healthy volunteers were also enroled as validation analysis. Patient characteristics are shown in Supplementary Table S2.

### RNA-seq analysis

RNA-seq analysis was performed against cancer cell lines treated with/without 5- Aza-CdR. Total RNA was isolated using an RNeasy Plus Mini Kit (QIAGEN); cDNA was synthesized using a ReverTra Ace qPCR RT Kit (Toyobo, Tokyo, Japan). Libraries for RNA-seq were prepared using the SureSelect Strand-Specific RNA Library Preparation Kit (Agilent Technologies, Santa Clara, CA) and subjected to massively parallel sequencing with a GAIIx (Illumina, San Diego, CA) using a single-end 36-bp or 50-bp sequencing length protocol. Sequenced tags were aligned to the human reference genome (build hg19) using CASAVA 1.8.2 (Illumina), and gene expression was normalized to the amount of reads per kilobase of exon per million mapped (rpkm) (Fig. 1a).

### DNA extraction and bisulfite modification

DNA extraction from fresh tissue was performed using a QIAamp DNA Mini Kit (QIAGEN, Hilden, Germany). Sodium bisulphite conversion was performed using the MethylEasy Xceed rapid DNA bisulfite modification kit (TaKaRa, Tokyo, Japan) following the manufacturer’s protocol.

### RRBS

Total 500 ng genomic DNA extracted from the cells were first digested with MspI restriction enzyme. DNA fragments were then subjected to end-repair and dA-tailing using an NEBNext DNA Library Prep Master Mix Set (NEB, Ipswich, MA), followed by the ligation with methylated adaptor oligo-DNA (NEB). After the size selection and the bisulfite conversion from unmethylated cytosine to uracil by using an EZ DNA Methylation kit (Zymo Research, Irvine, CA), DNA fragments were amplified by 18 cycles of PCR using primers included in the NEBNext kit. The generated library was sequenced by the 36 bp single-end protocol of GAIIx (Illumina). As unmethylated cytosine of the library is converted to thymine during the PCR amplification, mapping of sequenced tags to human reference genome (hg19, UCSC Genome Browser) was performed by Bismark software [42]. In this study, methylation level was estimated in CpGs with ≥10 reads (713,353 CpGs per sample in an average). Statistical analysis was done by methyl kit [43]. The promoters were defined as regions spanning −2.5 to +0.5 Kb from transcription start sites and containing ≥5 CpGs, and those being read ≥10 times were subjected to differential methylation analysis (Fig. 1a).

### Q-MSP

Aberrant DNA methylation, which often occurs around the transcriptional start site (TSS) within a CpG island, was evaluated using Q-MSP. The sequences of primers used in this study are shown in Supplementary Table S3. A standard curve for Q-MSP was constructed by plotting five serially diluted standard solutions of EpiScope methylated HeLa gDNA (TaKaRa). To analyse the normalised methylation value (NMV) of the PCR conditions, an analytical method was used as previously described [44].

### Detection of high-risk HPV DNA using PCR and p16 immunostaining

HPV status was determined using the HPV Typing Set (Takara), a primer set for PCR specifically designed to identify HPV genotypes 16, 18, 31, 33, 35, 52, and 58 using primary tumor DNA. The PCR HPV Typing Set method was performed according to the manufacturer’s instructions. To identify the HPV status of ctDNA, samples were also subjected to PCR using specific primers for HPV type 16. The primers for the HPV16 E7 sequence detection were 5′-TCCAGCTGGACAAGCAGAAC-3′ (forward primer), 5′-CACAACCGAAGCGTAGAGTC-3′ (reverse primer) [45]. For the immunohistochemistry analysis, an anti-human p16 monoclonal antibody (clone: E6H4, Roche Diagnostics GmbH, Germany) was used (Supplementary Fig. S1).

### Data analysis and statistics

A receiver operator characteristic (ROC) curve analysis of the target genes was performed with the NMVs for 79 matched paired HNSCC and normal mucosal samples using the Stata/SE 13.0 system (Stata Corporation, College Station, TX, USA). To determine the area under the ROC curve, the true positive rate (sensitivity) was plotted as a function of the false positive rate (1–specificity) for different cut-off points, and the NMV thresholds were calculated for each target gene. Cut-off values showing the greatest accuracy were determined based on sensitivity/specificity, as indicated in Supplementary Table S4. The methylation index was defined as the number of genes with promoter methylation [46]. Student’s *t* tests were performed to evaluate the associations between the clinical variables and the methylation index. DFS was investigated using the Kaplan–Meier method and the log-rank test. The probability of survival can be evaluated by generating a Kaplan–Meier curve. A Cox’s proportional hazards regression analysis that included age (≥65 vs. <65 years), gender, alcohol intake, smoking status, and tumour stage (I–II vs. III–IV) and methylation status was used to identify the multivariate predictive value of prognostic factors. A value of *P* < 0.05 was considered statistically significant.

## Supplementary information

Supplementary Fig. S1. p16 immunohistochemistry analysis of liquid biopsy patients under study.

Supplementary Table S1. Clinicopathological data of HNSCC patients under study.

Supplementary Table S2. Clinicopathological data of liquid biopsy patients under study.

Supplementary Table S3. Q-MSP Primer List

Supplementary Table S4. Results of the ROC curve analysis, the sensitivity, specificity, and cut off value.

Supplementary figure legends
