# Supplementary figures and images for: Identification of novel methylation markers in HPV-associated oropharyngeal cancer: genome-wide discovery, tissue verification and validation testing in ctDNA
Source: Oncogene. 2020 May 15;39(24):4741–55. doi: 10.1038/s41388-020-1327-z (PMC7286817; doi:10.1038/s41388-020-1327-z)

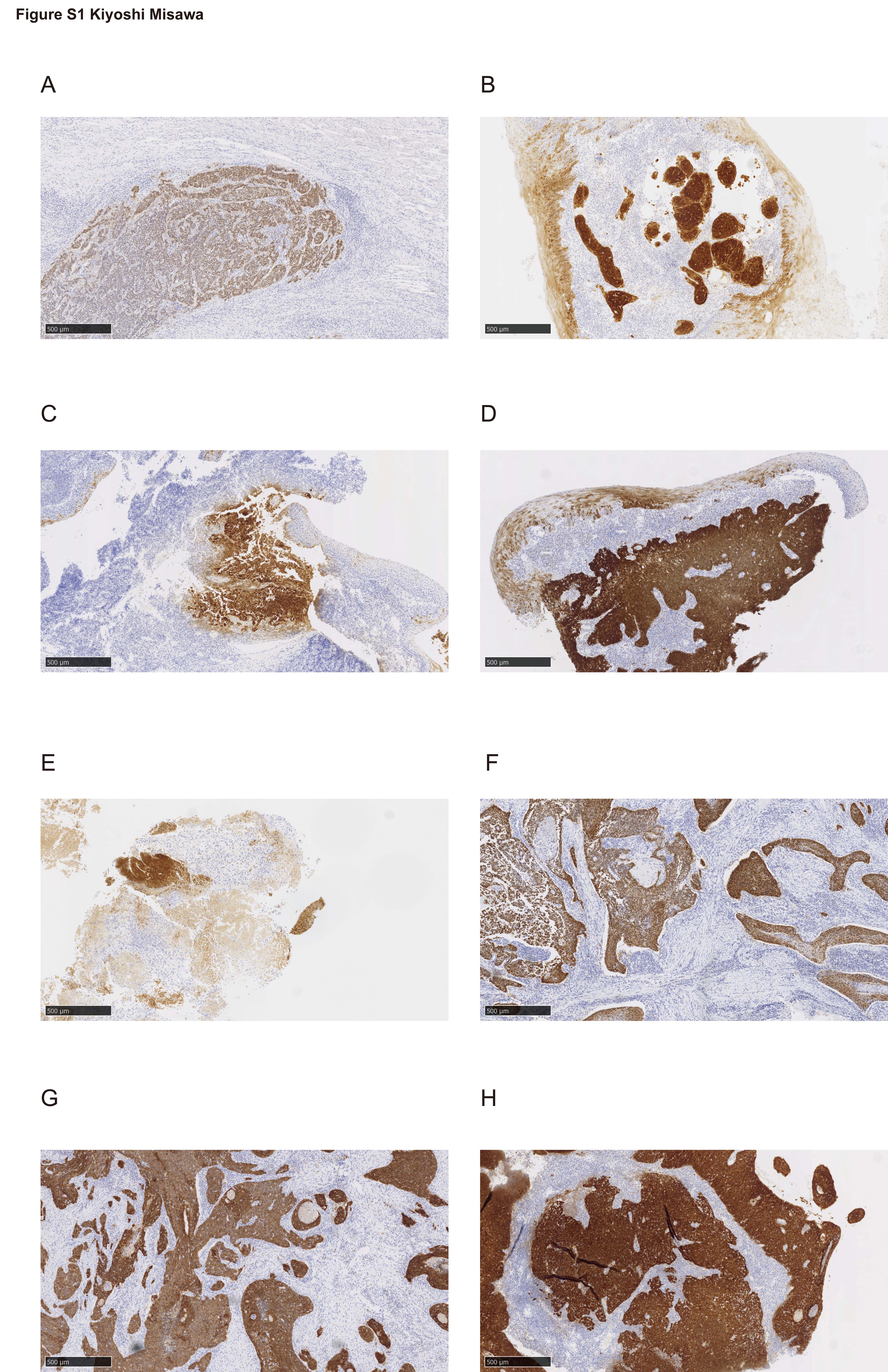

Supplement: Supplementary file 1 — Supplementary Fig. S1. p16 immunohistochemistry analysis of liquid biopsy patients under study. [file 41388_2020_1327_MOESM1_ESM.jpg]
