# Supplementary material for: Identification of novel methylation markers in HPV-associated oropharyngeal cancer: genome-wide discovery, tissue verification and validation testing in ctDNA
Source: Oncogene. 2020 May 15;39(24):4741–55. doi: 10.1038/s41388-020-1327-z (PMC7286817; doi:10.1038/s41388-020-1327-z)
Supplement: Supplementary file 2 — Supplementary Table S1. Clinicopathological data of HNSCC patients under study. [file 41388_2020_1327_MOESM2_ESM.docx]

| **Supplementary Table S1. Clinicopathological data of HNSCC patients under study.** | | | | | | | |
| --- | --- | --- | --- | --- | --- | --- | --- |
| Patient and tumor characteristics | | Full panel (n = 252) | Oropharyngeal cancer | | Hypopharyngeal cancer (n = 60) | Laryngeal cancer (n = 49) | Oral cavity cancer (n = 73) |
|  |  |  | HPV-associated (n = 35) | HPV-negative (n = 35) |  |  |  |
| *Age* | |  |  |  |  |  |  |
|  | Mean ± S.D. | 65.0 ± 10.9 | 63.0 ± 9.7 | 63.1 ± 10.4 | 67.1 ± 9.7 | 69.2 ± 8.0 | 62.3 ± 13.2 |
| *Gender* | |  |  |  |  |  |  |
|  | Female | 35 (13.9%) | 8 (22.9%) | 3 (8.6%) | 8 (13.3%) | 2 (4.1%) | 14 (19.2%) |
|  | Male | 200 (79.4%) | 27 (77.1%) | 32 (91.4%) | 52 (86.7%) | 47 (95.9%) | 59 (80.8%) |
| *Smoking status* | |  |  |  |  |  |  |
|  | Ever | 191 (75.8%) | 20 (57.1%) | 28 (80.0%) | 47 (78.3%) | 44 (89.8%) | 52 (71.2%) |
|  | Never | 61 (24.2%) | 15 (42.9%) | 7 (20.0%) | 13 (21.7%) | 5 (10.2%) | 21 (28.8%) |
| *Alcohol exposure* | |  |  |  |  |  |  |
|  | Ever | 191 (75.8%) | 25 (71.4%) | 31 (88.6%) | 52 (86.7%) | 34 (69.4%) | 49 (67.1%) |
|  | Never | 61 (24.2%) | 10 (28.6%) | 4 (11.4%) | 8 (13.3%) | 15 (30.6%) | 24 (32.9%) |
| *Tumor size* | |  |  |  |  |  |  |
|  | T1 | 30 (11.9%) | 9 (25.7%) | 6 (17.1%) | 1 (1.7%) | 3 (6.1%) | 11 (15.1%) |
|  | T2 | 96 (38.1%) | 19 (54.3%) | 10 (28.6%) | 22 (36.7%) | 7 (14.3%) | 38 (52.1%) |
|  | T3 | 48 (19.0%) | 3 (8.6%) | 3 (8.6%) | 18 (30.0%) | 19 (38.8%) | 5 (6.8%) |
|  | T4 | 78 (31.0%) | 4 (11.4%) | 16 (45.7%) | 19 (31.7%) | 20 (40.8%) | 19 (26.0%) |
| *Lympho-node status* | |  |  |  |  |  |  |
|  | N0 | 103 (40.9%) | 10 (28.6%) | 10 (28.6%) | 17 (28.3%) | 26 (53.1%) | 40 (54.8%) |
|  | N+ | 149 (59.1%) | 25 (71.4%) | 25 (71.4%) | 43 (71.7%) | 23 (46.9%) | 33 (45.2%) |
| *Stage* | |  |  |  |  |  |  |
|  | I | 21 (8.3%) | 4 (11.4%) | 4 (11.4%) | 0 (0.0%) | 3 (6.1%) | 10 (13.7%) |
|  | II | 39 (15.5%) | 4 (11.4%) | 2 (5.7%) | 9 (15.0%) | 4 (8.2%) | 20 (27.4%) |
|  | III | 48 (19.0%) | 5 (14.3%) | 2 (5.7%) | 16 (26.7%) | 14 (28.6%) | 11 (15.1%) |
|  | IV | 144 (57.1%) | 22 (62.9%) | 27 (77.1%) | 35 (58.3%) | 28 (57.1%) | 32 (43.8%) |
| *Recurrence events* | |  |  |  |  |  |  |
|  | Positive | 132 (52.4%) | 19 (54.3%) | 13 (37.1%) | 34 (56.7%) | 29 (59.2%) | 37 (50.7%) |
|  | Negative | 120 (47.6%) | 16 (45.7%) | 22 (62.9%) | 26 (43.3%) | 20 (40.8%) | 36 (49.3%) |

S.D.: standard deviation
