# Supplementary material for: Identification of novel methylation markers in HPV-associated oropharyngeal cancer: genome-wide discovery, tissue verification and validation testing in ctDNA
Source: Oncogene. 2020 May 15;39(24):4741–55. doi: 10.1038/s41388-020-1327-z (PMC7286817; doi:10.1038/s41388-020-1327-z)
Supplement: Supplementary file 3 — Supplementary Table S2. Clinicopathological data of liquid biopsy patients under study. [file 41388_2020_1327_MOESM3_ESM.docx]

|  | **Supplementary Table S2. Clinicopathological data of liquid biopsy patients under study.** | | | | | | | | | | | | |
| --- | --- | --- | --- | --- | --- | --- | --- | --- | --- | --- | --- | --- | --- |
| Patient number | | Age (years) | Sex | Primary HPV status | P16  status | ctDNA HPV status | Smoking status | Alcohol exposure | TNM stage | Primary site | Initial treatment | DFS (months) | OS (months) |
| 1-1-L | | 42 | M | positive | positive | positive | Ever | Ever | T4N2M0 | R-Tonsil | oropharyngectomy, B-ND | 12 | 27 |
| 2-1-L | | 72 | F | positive | positive | negative | Ever | Ever | T4N2M0 | R-Tonsil | CRT (CDDP+70Gy) | 6 | 6 |
| 3-1-L | | 45 | M | positive | positive | negative | Ever | Ever | T4N0M0 | BOT | oropharyngectomy, B-ND | 12 | 12 |
| 4-1-L | | 73 | F | positive | positive | negative | Never | Ever | T2N2M0 | L-Tonsil | oropharyngectomy, L-ND | 22 | 27 |
| 5-1-L | | 66 | F | positive | positive | positive | Never | Never | T2N0M0 | L-Tonsil | oropharyngectomy, L-ND | 25 | 25 |
| 6-1-L | | 47 | M | positive | positive | positive | Never | Never | T2N1M0 | L-Tonsil | oropharyngectomy, L-ND | 4 | 13 |
| 7-1-L | | 60 | M | positive | positive | positive | Ever | Ever | T2N2M0 | L-Tonsil | oropharyngectomy, B-ND | 12 | 12 |
| 8-1-L | | 61 | M | positive | positive | positive | Ever | Ever | T2N1M0 | R-Tonsil | CRT (CDDP+66Gy) | 12 | 12 |
|  | DFS, disease-free survival; OS, overall survival; M, Male; F, Female; BOT, base of tongue; ND, neck dissection; CRT, chemoradiotherapy; B, bilateral; R, right; L, left. | | | | | | | | | | | | |
