# Supplementary material for: Identification of novel methylation markers in HPV-associated oropharyngeal cancer: genome-wide discovery, tissue verification and validation testing in ctDNA
Source: Oncogene. 2020 May 15;39(24):4741–55. doi: 10.1038/s41388-020-1327-z (PMC7286817; doi:10.1038/s41388-020-1327-z)
Supplement: Supplementary file 4 — Supplementary Table S3. Q-MSP Primer List [file 41388_2020_1327_MOESM4_ESM.docx]

| **Supplementary Table S3. Q-MSP Primer List** | |  |
| --- | --- | --- |
| Gene | Forward primer 5’-3’ | Reverse primer 5’-3’ |
| ACTB | GGGTGGTGATGGAGGAGGTT | TAACCACCACCCAACACACAAT |
| AGAP2 | CGTCGGTGTTAGAGGTTTCGA | GCGCTACTCGTACTAATCCAC |
| ALPL | TTCGCGGTCGTTTTTATAAGG | TCCGTCCAAACACAAACGCGT |
| ANGPTL2 | GTACGCGGTTGTACGTATAGT | CAAACAACTCCCGCGTACTACG |
| ATP2A1 | GCGGAATTTGGAGAAATACGG | CGCGTACATACGTATACGTAT |
| CALML5 | TTCGCGAGGTGTAGGAGACG | CCGTTTCCATCCGTATCAACCG |
| DNAJC5G | GCGCGCGAAATAAGTTTTAGG | CTTATACGCAACGTCGACGCT |
| FDFT1 | TGAGAGTCGCGTTCGGGAGTT | TCCGCTTACCCCCGATCCGAA |
| GNMT | TGCGGAGCGGGTGGTTGCGGA | CCGAAAACCCTTCGACCGCC |
| GPT | CGAGTATAGGTGATCGGAGTTAGGC | GCACTACGTACTCCACTCTCCG |
| HOXB3 | GGTTCGGCGTGTTACGTGATT | ACAACCGCGACGTTATCGTAA |
| KLK11 | CGTTCGGGTTTCGTAGATGTA | CGCTTCTCCCTACCGCCCAAA |
| LMF1 | GCGGGGTTTTCGAGAATGGGC | CGAACGCCGCCATTATTAAAC |
| LY6D | AACGGACGGTAGCGAGAGAAT | CTCCGCAACGAAACCCAATAA |
| LYNX1 | GTAGCGAGGCGGGTGCGTTAA | CGTTTATCGACCGACACCAAC |
| MAL | GCGTAGTATTAAGTAGAGAGGTTCG | AATAAAAAATAAAACCGACCGC |
| MGC16275 | CGGTTTGGGGCGGCGTAAATT | ACTACACAACGTCCCGCACGT |
| MRGPRF | AAGGTTTCGGGTCGCGTTCGG | CTAATCCGCCGAAACCTCCTT |
| NKPD1 | CGTATTAGGTTTCGTTTATTTC | ACAACTCCTCGCTCTCGCGCT |
| SH2D3C | TAGTTTTCGTTTTCGGAGTTC | CCGCCGACAACCCGAAAACCT |
| TNNI2 | TAGCGCGTTATCGAGTTTTAA | TCCGACCGCCTAACCTAACCG |
| ZNF876P | GGCGTCGTTTTTGCGTTAGGT | GACGAAAACTAAAACTATAACCG |
