# Supplementary material for: Identification of novel methylation markers in HPV-associated oropharyngeal cancer: genome-wide discovery, tissue verification and validation testing in ctDNA
Source: Oncogene. 2020 May 15;39(24):4741–55. doi: 10.1038/s41388-020-1327-z (PMC7286817; doi:10.1038/s41388-020-1327-z)
Supplement: Supplementary file 5 — Supplementary Table S4. Results of the ROC curve analysis, the sensitivity, specificity, and cut off value. [file 41388_2020_1327_MOESM5_ESM.docx]

| **Supplementary Table S4. Results of the ROC curve analysis, the sensitivity, specificity, and cut off value.** | | | | |
| --- | --- | --- | --- | --- |
| Genes | ROC Area | Sensitivity (%) | Specificity (%) | Cutoff value |
| AGAP2 | 0.5428 | 34.0 | 90.0 | 0.3100 |
| ALPL | 0.5012 | 8.0 | 96.0 | 0.0020 |
| ANGPTL2 | 0.4928 | 38.0 | 82.0 | 0.0515 |
| ATP2A1 | 0.5120 | 24.0 | 98.0 | 0.0911 |
| CALML5 | 0.5156 | 34.0 | 89.4 | 0.3279 |
| DNAJC5G | 0.5898 | 44.0 | 84.0 | 0.1011 |
| FDFT1 | 0.5592 | 20.0 | 90.0 | 0.4700 |
| GNMT | 0.5092 | 22.0 | 98.0 | 0.0490 |
| GPT | 0.5504 | 30.0 | 90.0 | 0.1550 |
| HOXB3 | 0.5560 | 52.0 | 66.0 | 0.1340 |
| KLK11 | 0.6090 | 37.5 | 88.5 | 0.2488 |
| LMF1 | 0.5308 | 12.0 | 96.0 | 0.0001 |
| LY6D | 0.5666 | 34.7 | 88.2 | 0.0687 |
| LYNX1 | 0.5470 | 24.5 | 96.1 | 0.3731 |
| MAL | 0.4861 | 27.1 | 91.7 | 0.2981 |
| MGC16275 | 0.7321 | 51.0 | 84.3 | 0.0170 |
| MRGPRF | 0.6412 | 40.0 | 92.0 | 0.1714 |
| NKPD1 | 0.5289 | 27.1 | 89.6 | 0.1845 |
| SH2D3C | 0.5304 | 10.4 | 95.8 | 0.0058 |
| TNNI2 | 0.4569 | 24.5 | 89.8 | 0.256 |
| ZNF876P | 0.6597 | 55.1 | 81.6 | 0.0393 |
