# Supplementary material for: Identification of novel methylation markers in HPV-associated oropharyngeal cancer: genome-wide discovery, tissue verification and validation testing in ctDNA
Source: Oncogene. 2020 May 15;39(24):4741–55. doi: 10.1038/s41388-020-1327-z (PMC7286817; doi:10.1038/s41388-020-1327-z)
Supplement: Supplementary file 6 — Supplementary figure legends [file 41388_2020_1327_MOESM6_ESM.docx]

**Supplementary figure legends**

**Supplementary Fig. S1.** **p16 immunohistochemistry analysis of liquid biopsy patients under study.** (A) 1-1-L, (B) 1-2-L, (C) 1-3-L, (D) 1-4-L, (E) 1-5-L, (F) 1-6-L, (G) 1-7-L, and (H) 1-8-L.

**Supplementary Table S1. Clinicopathological data of HNSCC patients under study.** S.D.: standard deviation

**Supplementary Table S2. Clinicopathological data of liquid biopsy patients under study.** DFS, disease-free survival; OS, overall survival; M, Male; F, Female; BOT, base of tongue; ND, neck dissection; CRT, chemoradiotherapy; B, bilateral; R, right; L, left.

**Supplementary Table S3. Q-MSP Primer List**

**Supplementary Table S4. Results of the ROC curve analysis, the sensitivity, specificity, and cut off value.**
